# Altered functional connectivity density in the prefrontal-limbic-visual networks of vestibular migraine

**DOI:** 10.1038/s41598-026-38116-3

**Published:** 2026-02-10

**Authors:** Xia Zhe, Xiaoling Zhang, Miao Cheng, Min Tang, Xin Zhang, Xiaoyan Lei

**Affiliations:** 1 Department of MRI, Shaanxi Provincial People’s Hospital, Xi’an, 710068 Shaanxi China; 2 Department of Neurology, Shaanxi Provincial People’s Hospital, Xi’an, 710068 Shaanxi China

**Keywords:** Vestibular migraine, Functional connectivity density, Resting-state fMRI, Prefrontal cortex, Precuneus, Dizziness handicap inventory, Diseases, Medical research, Neurology, Neuroscience

## Abstract

This study aimed to explore abnormal patterns of functional connectivity density (FCD) and functional connectivity (FC) in patients with vestibular migraine (VM) and their associations with clinical symptoms. Resting-state functional magnetic resonance imaging (rs-fMRI) data from 49 VM patients and 61 healthy controls (HCs) were analyzed using Global FCD (GFCD), long-range FCD (LRFCD), and seed-based FC. Compared with HCs, VM patients demonstrated decreased GFCD and LRFCD in the bilateral medial prefrontal cortex (mPFC), along with increased GFCD in the right lingual gyrus (LING), right middle occipital cortex (MOC), left precuneus (preCUN), and elevated LRFCD in the middle cingulate cortex (MCC) and bilateral MOC. Seed-based FC analysis revealed significantly reduced connectivity between the mPFC and multiple regions, including the right cuneus/precuneus (CUN/preCUN), bilateral posterior cingulate cortex (PCC), bilateral hippocampus/parahippocampus (HIPP/ParaHIPP), and left calcarine cortex (CAL) in VM patients. Correlation analysis identified a positive association between GFCD in the left preCUN and Dizziness Handicap Inventory (DHI) scores (*r* = 0.370, *p* = 0.011). These findings highlight disrupted prefrontal-limbic-visual network integration in VM, with precuneus dysfunction potentially linked to dizziness severity. This study provides novel insights into the neural mechanisms underlying VM, highlighting the role of altered functional integration in symptom manifestation.

## Introduction

Vestibular migraine (VM) is a condition that combines the features of migraine with vestibular disturbances, resulting in symptoms such as dizziness, vertigo, headache, nausea, and hypersensitivity to light and sound^1–3^. Affecting 2.7% of the population, VM is more prevalent in women, particularly during their reproductive years^4,5^. This condition significantly impacts patients’ daily lives, leading to disruptions in work, social activities, and overall well-being^2,4,6^. The complex nature of VM involves both neural and physiological dysfunctions within the central vestibular system, yet the precise mechanisms underlying these symptoms remain unclear and continue to be the focus of ongoing research.

Extensive neuroimaging studies using positron emission tomography (PET) and perfusion magnetic resonance imaging (MRI) have revealed widespread cortical hypermetabolism and hyperperfusion in patients with VM during rest^7,8^. Additionally, morphometric studies based on structural MRI have also documented significant reductions in gray matter volume^9–12^and cortical thickness^13^ in VM patients. Recent resting-state research has also shown that individuals with VM exhibit changes in the amplitudes of low-frequency fluctuations (ALFF) and regional homogeneity (ReHo) in brain regions involved in vestibular control and multisensory integration, such as the temporal lobe and cerebellum^14,15^. Several studies focusing on functional connectivity (FC) have reported significant alterations in FC across multiple brain regions in patients with VM. These regions include the anterior cingulate/midcingulate cortex^16–18^, middle frontal gyrus (MFG)^18,19^, precuneus^18,19^, insular cortex^18,19^, supplementary motor area^18^, superior frontal gyrus^14^, inferior frontal gyrus^19^, and temporal lobe^20^. These studies^14,16–20^ suggest the abnormal FC in specific brain regions and networks, helping to elucidate the pathophysiological mechanisms underlying VM. However, a systematic, data-driven assessment of functional hubs—brain regions with critically altered connectivity centrality across the entire brain—remains lacking. Identifying such hubs is essential, as the integration of vestibular, visual, and nociceptive signals, a core deficit in VM, likely depends on the integrity of these pivotal network nodes.

Functional connectivity density (FCD) is a reproducible, data-driven method that combines voxel FC and graph theory analysis to help better characterize the brain functional topological organization^21,22^. At its core, FCD quantifies the number of functional connections each voxel has with the rest of the brain, providing a measure of degree centrality. By calculating global FCD (GFCD), local FCD (lFCD), and long-range FCD (LRFCD), the FCD analysis achieves a multi-perspective evaluation of the entire brain. The GFCD reflects the functional coupling of the entire brain, lFCD exhibits local changes, and LRFCD exhibits functional integration between non-adjacent voxels^21,22^. Therefore, multi-perspective evaluation can provide more details about the FC of the brain region through this method. Brain regions with higher FCD values are considered to be closely interconnected hub regions that are more important for neural convergence and global information integration^22,23^. In the presence pathological changes in brain function and structure, FCD provide information about key network hubs, offering insights **crucial for** uncovering the neural mechanisms underlying disease onset^24,25^. Previous studies have showed that the efficient and orderly functioning of the brain depends precisely on local and remote connections^23,24,26,27^. The FCD method has been successfully employed to identify abnormal functional hubs in neuropsychiatric disorders, such as premenstrual syndrome^27^, postmenopausal females^25^, mild cognitive impairment^28^ and stroke^24^. The brain network changes in VM patients may lead to abnormal global communication and integration function^29^. However, to our knowledge, no research has been conducted on the changes in FCD in patients with VM.

Thus, the aims of our study were (1) to examine global and local alterations in spontaneous neural activity using resting-state fMRI with FCD analysis; (2) to assess the FC changes between the seed points and the whole brain voxels base on these brain regions showing FCD difference as seed points; and (3) to investigate the association between long- and short-range FCD values and clinical parameters and scales in patients with VM. Through this study, we seek to enhance the understanding of the neural mechanisms underlying VM and identify potential neuroimaging biomarkers for the condition.

## Materials and methods

### Participants

Forty-nine patients diagnosed with VM were recruited at Shaanxi Provincial People’s Hospital, China, between January 2023 and April 2025. Inclusion criteria for the VM group were : (1) a confirmed VM diagnosis by a neurologist following the International Classification of Headache Disorders, 3rd edition^1,2^; (2) age between 18 and 50 years; (3) right-handedness as assessed by the Edinburgh Handedness Inventory; (4) a documented history of at least five VM episodes within the past year; and (5) absence of regular VM medications use for a minimum of three days prior to undergoing fMRI scanning. To ensure a consistent study sample, participants were excluded if they had: (1) any coexisting neurological disorders (e.g., epilepsy, multiple sclerosis); (2) psychiatric conditions such as anxiety or depression that could affect outcomes, as determined by a neurologist through clinical interview based on DSM-5 criteria^30^ and, for a subset of later-recruited participants, supplemented by scores below clinical thresholds on the Hamilton Anxiety Rating Scale (HAMA) and Hamilton Depression Rating Scale (HAMD); (3) chronic non-VM-related pain syndromes; (4) alternative vestibular diagnoses (e.g., Meniere’s disease, cerebrovascular incidents); (5) a history of substance abuse, determined through self-report questionnaires; or (6) contraindications to MRI, including claustrophobia or metallic implants. All participants in the VM group underwent a standardized screening protocol consisting of: (1) MRI imaging to exclude structural abnormalities and vascular lesions, (2) neurological evaluation by a qualified neurologist, and (3) comprehensive neuro-otological assessments, which included videonystagmography, caloric testing, head impulse tests, and vestibular-evoked myogenic potential recordings. These tests were administered during symptom-free intervals to rule out peripheral vestibular involvement.

The healthy control (HC) group comprised 61 age-, sex-, and handedness-matched individuals from the local community. Control participants were screened to exclude any prior history of neurological, psychiatric, or vestibular conditions that could confound the results. The study was received approval form the Ethics Committee of Shaanxi Provincial People’s Hospital (Approval Code: [2023R120]). Written informed consent was obtained from all participants. All procedures adhered to ethical guidelines and were conducted in compliance with the Declaration of Helsinki.

### Clinical assessment

The dizziness Handicap Inventory (DHI) was employed to assess the level of self-reported disability associated with dizziness and balance disturbances^31^. All participants were asked to rate the intensity of migraine and vertigo/imbalance symptoms on a Visual Analog Scale (VAS), which ranges from 0 to 10^32^. Furthermore, the severity of migraine symptoms was evaluated using the Head Impact Test-6 (HIT-6) additional insight.

### Imaging data acquisition

Imaging was performed using a 3.0 T scanner (Philips Ingenia, Best, Netherlands) with a 16-channel head coil. High-resolution 3D T1-weighted magnetization-prepared rapid-acquisition gradient echo imaging was used to cover the entire brain with 332 sagittal slices. The parameters for the T1-weighted- sequence as follows: a repetition time (TR) = 1900 ms, flip angle (FA) = 9°, inversion time (TI) = 900 ms, echo time (TE) = 2.26 ms, matrix size = 256 × 256, slice thickness = 1.00 mm (no gap between slices), and field of view (FOV) = 220 × 220 mm. For resting-state functional BOLD imaging, gradient echo-planar imaging was utilized with the following settings: TR = 2000 ms, TE = 30 ms, 34 slices, slice thickness = 4 mm (no gap), FOV = 230 × 230 mm, matrix size = 128 × 128, flip angle = 90°, and 200 volumes over 6.5 min. Participants were instructed to close their eyes, remain awake, and maintain a relaxed posture during the scan. Afterward, they were asked to confirm their alertness during the procedure.

### Data preprocessing

The rs-fMRI data was analyzed by the Data Processing & Analysis for Brain Imaging (DPABI, http://rfmri.org/dparbi) with Statistical Parametric Mapping, version 12 (SPM12, http://www.fil.ion.ucl.ac.uk/spm) running on MATLAB platform. The preprocessing steps included the following: the first five time points were removed; slice timing; head motion correction: excluded subjects from further analysis if the translation or rotation of head movement was > 3 mm or > 3° in any direction; the corrected images were spatial normalized to the Montreal Neurological Institute (MNI) space (EPI template with 3 × 3 × 3 mm3 voxel size); nuisance signal removal (including 24 head motion parameters, white matter, cerebrospinal fluid as covariates). Moreover, smoothing using a Gaussian kernel with a full-width at half maximum (FWHM) of 6 mm. Linear detrended removal and temporal bandpass filtering (0.01–0.1 Hz) were performed.

### FCD analysis

To evaluate the FCD mapping for each subject, we conducted Pearson correlation analyses to assess voxel-wise functional correlations^33^. For each seed voxel (x₀), its time series was correlated with that of every other voxel. Voxels exhibiting a correlation coefficient *R* > 0.6 with x₀ were considered functionally connected^21^. This threshold was chosen in line with established methodological guidelines^21^, wherein lower thresholds (< 0.4) tend to increase false positive rates and computational burden, while higher thresholds (> 0.7) may compromise sensitivity and reduce dynamic range in FCD maps. Therefore, *R* > 0.6 offers an optimal trade-off for detecting robust functional connectivity. Based on this criterion, individual maps of both local FCD (lFCD) and global FCD (GFCD) were subsequently generated within a grey matter (GM) mask ^〔22〕^.

The short-range FCD (lFCD) was derived using a “growing algorithm” as follows^〔21〕^: (1) functional connectivity between x₀ and each of its directly adjacent voxels (x_i_) was computed. Those neighbors with *R* > 0.6 were retained. (2) for each retained neighbor x_i_, voxels directly adjacent to x_i_ but not to x₀ (xⱼ) were identified. (3) If the connectivity between x₀ and xⱼ also exceeded *R* > 0.6, xⱼ was added to the short-range cluster. (4) This iterative search continued until no new voxels could be included, defining the short-range FCD as the size of this local functional cluster.

Long-range FCD (LRFCD) was then calculated by subtracting the short-range FCD from the global FCD, representing the number of functionally connected voxels that were not part of the local spatial neighborhood of x₀.

To improve normality and allow for standardization, both short- and long-range FCD values at each voxel were divided by the participant’s whole-brain mean FCD. Finally, the normalized FCD maps were spatially smoothed using a Gaussian kernel with a full-width at half-maximum (FWHM) of 6 mm.

### FC analysis

In the current study, regions of interest (ROIs) were defined as the overlapped brain areas demonstrating abnormal lFCD/LRFCD/GFCD of VM in the substantially analysis. Pearson’s correlation analyses were performed between the mean time series of ROI and other time series of all brain voxels. In order to improve the normality of the correlation coefficient, Fisher r-z transform was used to transform r-value mapping into FC mapping (z-value).

### Statistical analysis

Demographic and neuropsychological data of the two groups were calculated by SPSS software (version 22.0; IBM, Armonk, New York). Two independent samples t-test was adopted for normally distributed continuous variables. Categorical variables were compared by Pearson’s Chi-square test. Statistical significance thresholded for all comparisons were set at *p* < 0.05.

The two-sample test was applied to explore the differences of the standardized FCD values between VM patients and HCs, controlling for age, gender and education as covariates (Gaussian Random Field (GRF) correction, cluster-level *p* < 0.05, voxel-level *p* < 0.005, and two-tailed). No minimum cluster size threshold was applied beyond the GRF correction.

We further assessed seed-based FC using the overlapped brain regions (i.e., bilateral mPFC) showing altered GFCD and LRFCD as seed regions. Between-group differences in seed-based FC (zFC maps) were examined using two-sample t-tests within DPABI, with age, sex, and education included as covariates. Multiple comparisons were corrected using the GRF method (cluster-level *p* < 0.05, voxel-level *p* < 0.005, and two-tailed).

Pearson’s correlation was performed to reveal the association between the altered FCD values and clinical symptom changes in patients with VM. The significant threshold was set at *p* < 0.05.

## Results

### Demographic and neuropsychological data

A total of 110 subjects were enrolled in this study, including 49 VM patients and 61 matched HCs. Their demographic and neuropsychological data are summarized in Table 1. Compared with HCs, the VM patients exhibited significantly lower in education years (*p* = 0.01). There were no significant differences between the two groups.

**Table 1:** Clinical and demographic features for VM patients and HCs.

Features	VM	HCs	*p* value
Gender (M/F)	8/41	8/53	0.79^a^
Age (years)	37.06 ± 10.17	37.15 ± 11.42	0.97^b^
Education (years)	14.16 ± 3.15	15.62 ± 2.35	0.01^b^
Disease Duration (years)	8.47 ± 9.06	-	-
Episode Frequency (number)	5.76 ± 4.23	-	-
DHI score	52.55 ± 9.87	-	-
MIDAS score		-	-
VAS score	5.14 ± 2.26	-	-
HIT-6 score	55.35 ± 14.75	-	-

Note: Data are expressed in mean ± standard deviation.

VM, vestibular migraine; HC, healthy control; VAS, Visual Analog Scale (0 = no pain, 10 = worst possible pain); HIT-6, Headache Impact Test-6; DHI, Dizziness Handicap Inventory.

^a^ The *p* value was obtained by the two-tailed chi-squared test between the VM and HCs.

^b^ The *p* value was obtained by two sample t-tests between the VM and HCs.

### Imaging result

**Fig. 1:**
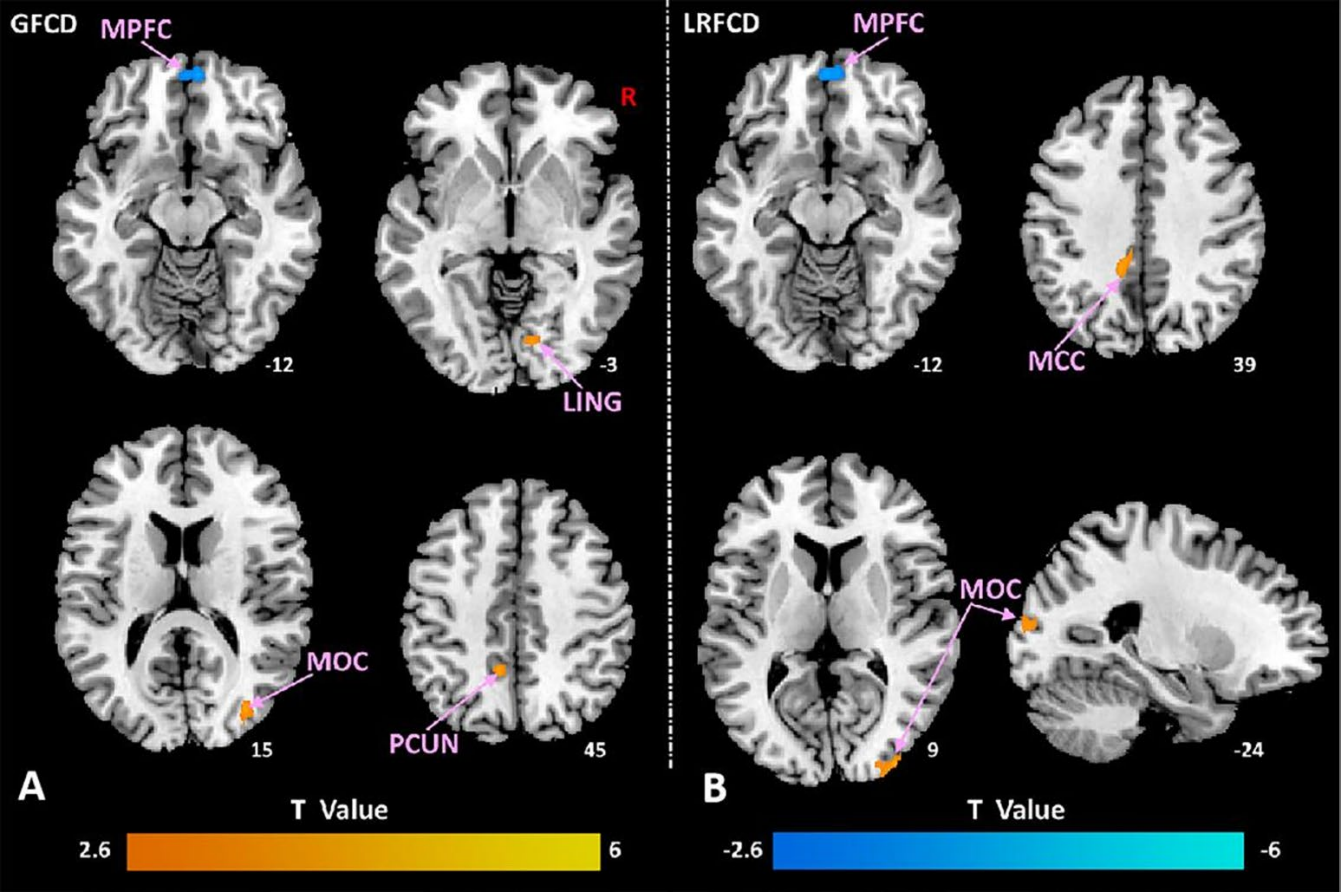
FCD alteration in patients with VM in contrast to HCs. **(A)** Axial slices showing brain regions with significant group differences in global FCD (GFCD). Compared to HCs, VM patients showed decreased GFCD in the bilateral medial prefrontal cortex (mPFC, blue clusters) and increased GFCD in the right lingual gyrus (LING), right middle occipital cortex (MOC), and left precuneus (preCUN, red clusters). **(B)** Axial and Sagittal slices showing group differences in long-range FCD (LRFCD). VM patients exhibited decreased LRFCD in the bilateral mPFC (blue) and increased LRFCD in the middle cingulate cortex (MCC) and bilateral MOC (red). All results are corrected for multiple comparisons (GRF correction, cluster-level *p* < 0.05, voxel-level *p* < 0.005, and two-tailed). Abbreviations are defined in the main text. The color bar represents the T-value from the two-sample t-test (red: VM > HC; blue: VM < HC). The numbers below each slice indicate the MNI Z-coordinate.

In comparison with HCs, VM patients had decreased GFCD and LRFCD in the bilateral medial prefrontal cortex (mPFC), increased GFCD in the right lingual gyrus (LING), right middle occipital cortex (MOC), left precuneus (preCUN), and increased LRFCD in the middle cingulate cortex (MCC), bilateral MOC (Fig. 1). No significant difference of LFCD was found between groups.

**Fig. 2:**
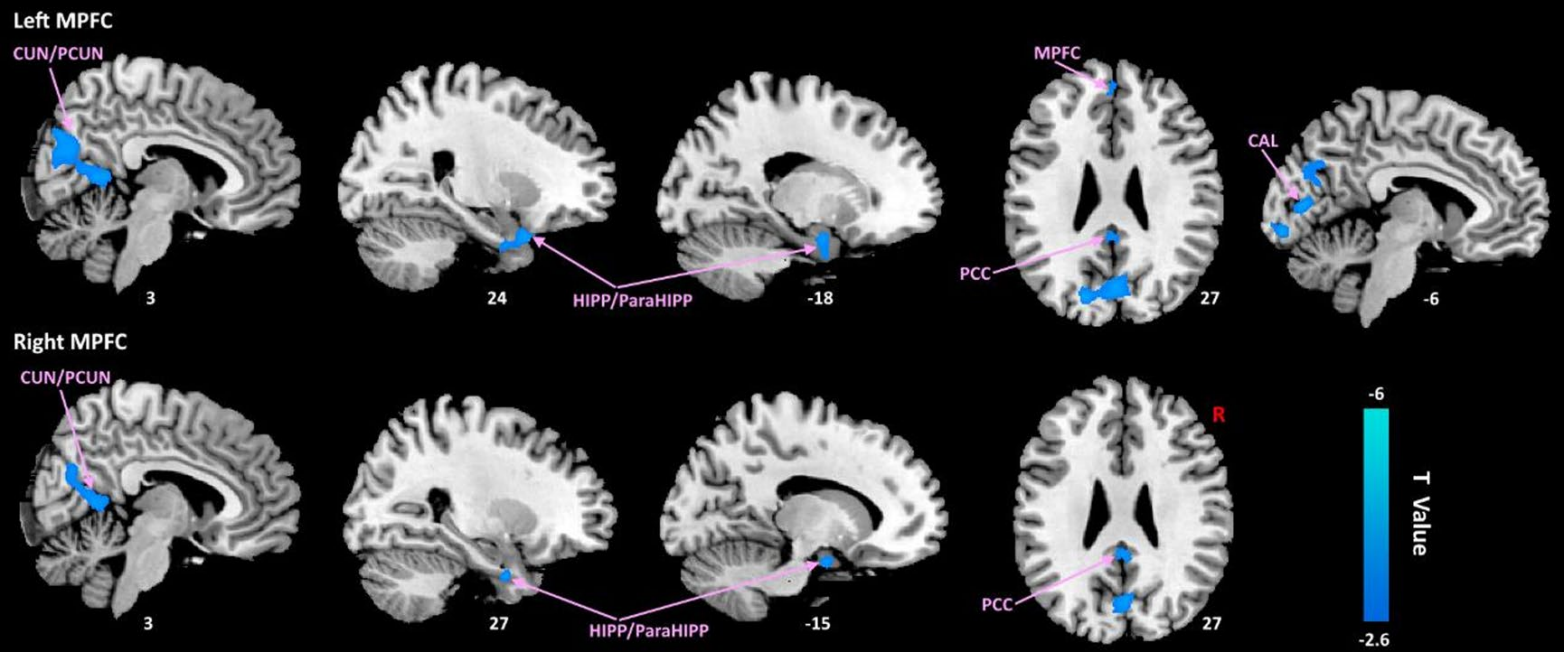
Abnormal mPFC-related FC of patients with VM in contrast to HCs.

In comparison with HCs, patients with VM showed remarkably lower resting-state FC between the mPFC and multiple brain regions, including the right cuneus/precuneus (CUN/preCUN), bilateral posterior cingulate cortex (PCC), bilateral hippocampus/parahippocampus (HIPP/ParaHIPP), bilateral cuneus, left calcarine (CAL) (Fig. 2).

The sagittal and axial views highlight reduced connectivity between the left mPFC and the right cuneus/precuneus (CUN/preCUN), bilateral posterior cingulate cortex (PCC), bilateral hippocampus/parahippocampus (HIPP/ParaHIPP), and left calcarine cortex (CAL).

Similarly, reduced FC was observed between the right mPFC seed and the left CUN/preCUN, bilateral PCC, and bilateral HIPP/ParaHIPP in VM patients. The color bar represents the T-value (blue: HC > VM). Results are displayed on a canonical brain surface and sections at the indicated MNI coordinates (X, Y, Z), corrected for multiple comparisons (GRF correction, cluster-level *p* < 0.05, voxel-level *p* < 0.005, and two-tailed).

### Correlation analysis result

**Fig. 3:**
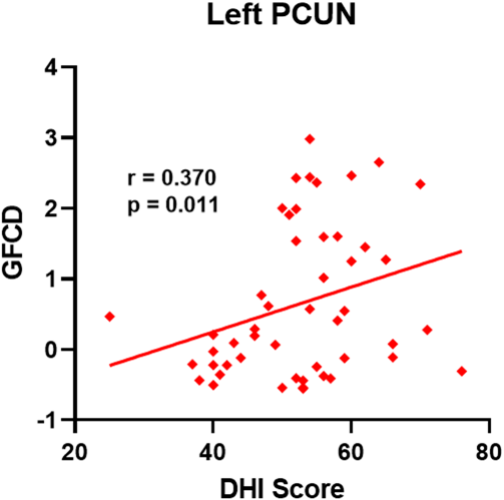
The positive correlation between the GFCD in the left PCUN and DHI scores in patients with VM.

Patients with VM showed a positive connection between GFCD in the left PCUN and the DHI score (*r* = 0.370, *p* = 0.011; Fig. 3).

## Discussions

In this study, we employed BOLD-fMRI to investigate alterations of FCD and seed-based FC in VM patients. The main findings were: (1) we found that, compared to HCs, VM patients had decreased GFCD in the bilateral mPFC, increased GFCD in the LING, MOC and PCUN, decreased LRFCD in the bilateral mPFC as well as increased LRFCD in the MCC and bilateral MOC; (2) Compared to HCs, VM patients showed decreased FC between the bilateral mPFC as ROIs and the right CUN/peCUN, bilateral HIPP/ParaHIPP, left mPFC, bilateral PCC, and left CAL; and (3) the positive correlation between the GFCD in the left PCUN was positively correlated with DHI scores in VM patients.

It was found that patients had significantly decreased LRFCD and GFCD in the bilateral mPFC of VM compared to HCs in this study, which was consistent with the one of the previous findings^29^. As a key node of the default mode network (DMN), the mPFC primarily participated in self-relevant cognitive processes and memory scene construction^34^, and was involved in modulating pain responses by inhibiting amygdala activity and regulating emotional states^35^. In the context of VM pathophysiology, the observed synchronous decline in LRFCD and GFCD in the mPFC may indicate a disruption in its top-down regulatory capacity. This could impair the integration of vestibular signals with cognitive and emotional contexts, potentially contributing to the maladaptive processing of vestibular stimuli and the exacerbation of symptoms like dizziness and headache.

To further explore the functional implications of this mPFC hub alteration, we conducted a whole-brain FC analysis using the bilateral mPFC clusters (identified by the data-driven FCD analysis) as seed regions. The results revealed that the altered FC in these patients was primarily concentrated in the DMN, specifically showing reduced FC between the mPFC and the cCUN/PCUN, HIPP/ParaHIPP, and PCC. The DMN consists of brain regions that exhibit strong functional connectivity during rest and is typically suppressed when attention is directed toward external stimuli. It is considered a critical network in the resting state, closely linked to cognitive functions such as memory integration, language, and semantic representation. The PCC, as another key node in the DMN, works in conjunction with the mPFC to engage in self-relevant cognitive activities and plays a significant role in spontaneous cognition^34^. Moreover, research has shown that in patients with migraines, the functional connectivity between the prefrontal cortex and the PCC is reduced^36^(4). The abnormal FC between the mPFC and PCC in VM patients may suggest a disruption in their functional coordination and information transfer. The precuneus is also implicated in pain regulation^37,38^ (5,6), with Philippe Goffaux et al. finding that although the precuneus does not directly perceive pain, it is closely associated with pain sensitivity^39^(7). The altered FC between the precuneus and mPFC may contribute to abnormal pain sensitivity in VM patients, potentially leading to atypical pain perception. These findings suggest that the reduced functional connectivity of the mPFC in VM patients may reflect a disruption in its role within the DMN, offering new neurobiological insights for understanding the cognitive and pain regulation processes in these patients.

In addition, the group with VM showed increased GFCD in the right lingual gyrus (LING), right middle occipital cortex (MOC), left precuneus (preCUN), and increased LRFCD in the middle cingulate cortex (MCC), bilateral MOC, compared with the HCs. These alterations suggest hyper-connectivity in regions associated with visual processing and attentional control, potentially reflecting compensatory mechanisms or maladaptive neural plasticity in response to chronic vestibular dysfunction. The occipital cortex, including the lingual gyrus and MOC, plays a central role in visual perception and spatial orientation^40–42^. Enhanced connectivity in these regions may indicate heightened visual dependence, a well-documented phenomenon in patients with vestibular disorders^43,44^. Previous studies have shown that VM patients tend to rely more heavily on visual cues for spatial orientation, potentially leading to visual-vestibular mismatch and exacerbated dizziness symptoms^45^. Our findings of increased GFCD and LRFCD in visual areas are in line with these prior observations, supporting the notion of altered visual network functioning in VM.

The precuneus (preCUN), part of the posterior default mode network, is involved in visuospatial imagery, self-referential thinking, and integration of multisensory information^46,47^. Its increased GFCD in VM may reflect an abnormal role in processing internal representations of body position and spatial context. Notably, we observed a significant positive correlation between GFCD in the left precuneus and Dizziness Handicap Inventory (DHI) scores, indicating that hyperconnectivity in this region may be associated with greater subjective dizziness severity. However, it is important to note that this correlation, while statistically significant (*p* = 0.011), explains only a portion of the variance, suggesting that other neurobiological and psychological factors also contribute to dizziness handicap in VM. This finding suggests that altered functional connectivity in the precuneus could be one component contributing to the distorted spatial perception and self-orientation often reported by patients with VM.

Increased LRFCD in the middle cingulate cortex (MCC) may also be relevant to the chronic and emotionally distressing nature of VM. The MCC is implicated in affective pain processing, attention modulation, and interoceptive awareness^48,49^. Its elevated connectivity may reflect heightened vigilance or salience attribution to internal vestibular and nociceptive signals. Prior fMRI research has linked increased MCC activity to maladaptive attentional control in chronic migraine and other somatosensory disorders^50,51^, suggesting a similar mechanism may be at play in VM. It is crucial to clarify that discussing the role of the MCC in affective processing does not imply our cohort had comorbid anxiety or depressive disorders (which were exclusion criteria). Rather, it points to the inherent illness-related emotional burden and salience of chronic, unpredictable vestibular symptoms, which can be distressing and provoke heightened interoceptive awareness even in the absence of a formal mood disorder diagnosis.

Together, these findings indicate that VM is not only characterized by reduced connectivity in frontal control hubs but also by hyperconnectivity in posterior and cingulate networks. This imbalance may reflect a shift in sensory weighting toward visual and interoceptive inputs, contributing to the clinical presentation of dizziness, visual sensitivity, and spatial disorientation.

### Limitations

Several limitations should be considered in this study. First, the cross-sectional design limits causal interpretations between altered functional connectivity and clinical symptoms. Second, the sample size, though moderate, may limit generalizability to broader VM populations. Third, potential confounding factors such as medication use or comorbid conditions were not fully controlled, which may influence brain connectivity patterns. Fourth, the relatively short acquisition time for the resting-state fMRI scans (6.5 min) is a potential limitation, as longer scan durations are generally recommended to improve the reliability of functional connectivity metrics. Fifth, susceptibility distortion correction was not applied during the preprocessing of the EPI data, which could theoretically influence spatial accuracy in regions prone to distortion. Finally, the correlation between precuneus GFCD and DHI, while significant, was modest and uncorrected for multiple comparisons; these results should be considered preliminary and require replication in independent cohorts.

## Conclusion

This study revealed altered functional connectivity density and disrupted network integration in patients with vestibular migraine, particularly involving the prefrontal, limbic, and visual regions. The association between precuneus hyperconnectivity and dizziness severity suggests a potential neural basis for subjective symptom burden. These findings contribute to a deeper understanding of VM pathophysiology and may inform future diagnostic or therapeutic strategies.

## Data Availability

To obtain the original data supporting the conclusions of this study, please reach out to the corresponding author.
